# Multiple myeloma-derived exosomes are enriched of amphiregulin (AREG) and activate the epidermal growth factor pathway in the bone microenvironment leading to osteoclastogenesis

**DOI:** 10.1186/s13045-018-0689-y

**Published:** 2019-01-08

**Authors:** Stefania Raimondo, Laura Saieva, Emanuela Vicario, Marzia Pucci, Denise Toscani, Mauro Manno, Samuele Raccosta, Nicola Giuliani, Riccardo Alessandro

**Affiliations:** 1 Department of Biopathology and Medical Biotechnologies (Di.Bi.Med.), University of Palermo, Palermo, Italy; 2 Department of Medicine and Surgery, University of Parma, Parma, Italy; 3 Institute of Biophysics (IBF), National Research Council (CNR) of Italy, Palermo, Italy

**Keywords:** Exosomes, Multiple myeloma, Bone disease, Epidermal growth factor receptor, Amphiregulin, Interleukin 8

## Abstract

**Background:**

Multiple myeloma (MM) is a clonal plasma cell malignancy associated with osteolytic bone disease. Recently, the role of MM-derived exosomes in the osteoclastogenesis has been demonstrated although the underlying mechanism is still unknown. Since exosomes-derived epidermal growth factor receptor ligands (EGFR) are involved in tumor-associated osteolysis, we hypothesize that the EGFR ligand amphiregulin (AREG) can be delivered by MM-derived exosomes and participate in MM-induced osteoclastogenesis.

**Methods:**

Exosomes were isolated from the conditioned medium of MM1.S cell line and from bone marrow (BM) plasma samples of MM patients. The murine cell line RAW264.7 and primary human CD14^+^ cells were used as osteoclast (OC) sources.

**Results:**

We found that AREG was specifically enriched in exosomes from MM samples and that exosomes-derived AREG led to the activation of EGFR in pre-OC, as showed by the increase of mRNA expression of its downstream *SNAIL* in both RAW264.7 and CD14^+^ cells. The presence of neutralizing anti-AREG monoclonal antibody (mAb) reverted this effect. Consequently, we showed that the effect of MM-derived exosomes on osteoclast differentiation was inhibited by the pre-treatment of exosomes with anti-AREG mAb. In addition, we demonstrated the ability of MM-derived AREG-enriched exosomes to be internalized into human mesenchymal stromal cells (MSCs) blocking osteoblast (OB) differentiation, increasing MM cell adhesion and the release of the pro-osteoclastogenic cytokine interleukin-8 (IL8). Accordingly, anti-AREG mAb inhibited the release of IL8 by MSCs suggesting that both direct and indirect effects are responsible for AREG-enriched exosomes involvement on MM-induced osteoclastogenesis.

**Conclusions:**

In conclusion, our data indicate that AREG is packed into MM-derived exosomes and implicated in OC differentiation through an indirect mechanism mediated by OBs.

**Electronic supplementary material:**

The online version of this article (10.1186/s13045-018-0689-y) contains supplementary material, which is available to authorized users.

## Background

Multiple myeloma (MM) is a clonal plasma cell (PC) malignancy characterized by the abnormal accumulation of malignant MM cells within the bone marrow (BM) which leads to osteolytic bone disease [1, 2]. Active MM is characterized by severe imbalance between osteoclasts (OCs), responsible for bone resorption and osteoblasts (OBs), responsible for bone formation [3]. The increased OC activity promotes the progression of MM, allowing the establishment of a loop between bone destruction and the survival of cancer cells [4].

MM cells stimulate OC differentiation mainly indirectly stimulating the production, by the surrounding cells, of soluble factors [5] such as the receptor activator of nuclear factor kappa-Β ligand (RANKL). Other cytokines produced directly by MM cells and their microenvironment as interleukins (ILs) IL-1β, IL-3, and IL-6 are also involved in MM-induced osteoclastogenesis [6]. The increased osteolytic activity induced by MM cells is strictly associated with the inhibition of OB differentiation in human mesenchymal stromal cells (MSCs), resulting in a marked alteration of bone remodeling. In MM, the presence of cytokines and the physical interaction between MSCs and MM cells impaired bone formation by inhibiting osteogenic precursors and stimulated the production of osteoclastogenic cytokines that support OC formation. [7, 8].

In recent years, several studies have been focused on the possible role of exosomes, nanosize lipoproteic structures that have been recognized as a new mechanism of cell-to-cell communication. This feature is attributed to their ability to actively transport mRNA, miRNA, proteins, and growth factors towards target cells, modifying their behavior as well as the microenvironment [9, 10].

Emerging evidence highlighted the role of exosomes in several cancer types including MM; cell-derived extracellular vesicles are in fact considered regulators of MM BM microenvironment and are able to promote MM progression in a paracrine and autocrine manner [11–15]. Furthermore, our previous data have shown that MM cell-derived exosomes play a relevant functional role in OC differentiation [16]. In particular, we found that exosome treatment increased the expression of OC-specific markers, such as tartrate-resistant acid phosphatase (TRAP), cathepsin K (CTSK), and matrix metallopeptidase 9 (MMP9) and directly control OC formation and activity. These results highlight the ability of exosomes to affect directly OC differentiation and function. Here, we focus on defining how MM cell-derived exosomes act on osteoclastogenesis.

The epidermal growth factor receptor (EGFR) is a transmembrane glycoprotein with intrinsic tyrosine kinase activity that can be bound and activated by a family of seven peptide growth factors including amphiregulin (AREG). The EGFR system acts in various cellular physiological and pathological processes such as proliferation, differentiation, and motility. Recent studies have reported that this signaling network plays an essential role in bone metabolism by affecting both OBs and OCs [17]. Further studies have revealed that EGFR ligands are able to stimulate OC formation by decreasing in OBs the expression of OPG [18]. In addition to the EGFR system, IL8, a cytokine well studied for its role in promoting tumor angiogenesis, cell motility, and invasion [19], has been described as an activator of bone destruction in metastatic and MM bone disease [20, 21].

Here, we investigated whether MM exosomes, from both cell lines and MM patients, affect OC differentiation by activating the EGFR pathway.

## Methods

### Data set analysis

The expression of AREG at mRNA level on PCs from 11 monoclonal gammopathy of undetermined significance, 133 MM patients at the diagnosis, 9 primary plasma cell leukemia (PCL), and 4 healthy donors (GSE16122) [22], generated on AffymetrixGeneChip HG-U133A arrays (Affymetrix, Santa Clara, CA, USA), were extracted from CEL files using RMA normalization procedure and custom CDF annotation package (GeneAnnot v2.2.1, Rehovot, Israel), as previously described [23].

### Reagents

Recombinant AREG (R&D Systems, Abingdon, UK) was reconstituted at 0.1 mg/ml in sterile PBS, aliquoted and stored at − 20 °C. Neutralizing anti-AREG mAb (R&D Systems, Abingdon, UK) was reconstituted at 0.2 mg/ml in sterile PBS, aliquoted and stored at − 20 °C. SB225002 (Cayman Chemical, MI, USA) was solubilized at 10 mM stock solution in DMSO and stored at 20 °C.

### Cells and cell culture conditions

#### Cell lines

The human myeloma cell line (HMCL) MM1.S was purchased from Leibniz Institute Deutsche Sammlung von Mikroorganismen und Zellkulturen GmbH (Braunschweig, Germany). Cells were maintained in RPMI-1640 medium supplemented with 10% fetal bovine serum (FBS), l-glutamine (2 mM), and antibiotics (100 U/ml penicillin and 100 μg/ml streptomycin), all obtained from ThermoFisher Scientific (Waltham, MA, USA).

Murine macrophage RAW 264.7 cells were purchased from ATCC (Manassas, VA, USA). Cells were cultured in Dulbecco’s modified Eagle’s medium (DMEM) supplemented with 10% FBS, 2 mM l-glutamine, 100 U/ml penicillin, and 100 μg/ml streptomycin (Euroclone, Milan, Italy) and differentiated to OC as previously described [24]. The human telomerase reverse transcriptase transduced mesenchymal stromal cell line (hTERT-MSCs) was kindly gifted by Dr. Giuseppe Gaipa (Monza, Italy). hTERT-MSCs were cultured in mesenchymal stem cell growth medium (MSCGM™ Bullet Kit, Lonza, Walkersville, MD, USA) to maintain them into an undifferentiated condition and in Mesenchymal Stem Cell Osteogenic Differentiation Medium to induce osteogenic differentiation (MSC Osteogenic Differentiation BulletKit™, Lonza).

#### CD14^+^ monocytes isolation

Human peripheral mononuclear cells (PBMCs) were isolated by Ficoll-Paque (GE Helthcare-Bio Science, Uppsala, Sweden) from whole blood of the healthy donors in accordance with the Declaration of Helsinki guidelines and University of Palermo Ethics committee. Once isolated, cells were washed with MACS isolation buffer for monocyte isolation. PBMCs were then incubated with human CD14 microbeads (MiltenyiBiotec, BergischGladbach, Germany) for 15 min at 4 °C. The magnetic separation was performed using LS columns (MiltenyiBiotec), and the bound cells were then washed and suspended in complete medium for further experiments.

#### Exosome isolation

Exosomes released by MM1.S after a 48h culture period in presence of FBS previously ultracentrifuged (exosome-free FBS) were isolated from conditioned culture medium by differential centrifugation, as previously described [25]. Briefly, culture medium was centrifuged subsequently for 5 min at 300×*g*, 15 min at 3000×*g*, and 30 min at 10,000×*g* and ultracentrifuged 90 min at 100,000×*g* in a Type 70 Ti, fixed angle rotor.

Exosomes were isolated from bone marrow (BM) plasma of four MM patients (three newly diagnosed and one relapsed). All patients provided written informed consent in accordance with the Declaration of Helsinki. The Institutional Review Board of the University of Parma (Italy) approved this part of the study. Exosomes were isolated from human plasma and prepared as described above. Exosome pellets were washed and suspended in PBS, and exosome protein content was determined by the Bradford assay.

#### Cell treatment

Exosomes (50 μg/ml) previously isolated from either MM1.S or BM plasma MM samples were treated or not with anti-AREG mAb (50 μg/ml) for 2 h at 37 °C. Both human primary CD14^+^ monocytes and RAW 264.7 cells were incubated for 3 and 6 days in osteoclastogenic medium (recombinant human (rh) RANKL 25 ng/ml and MCSF 25 ng/ml), with exosomes treated or not with anti-AREG mAb and with rhAREG (50 μg/ml). The media were changed every 3 days. At the end of the culture period, OC differentiation and EGFR activation were assessed as described below. Human primary CD14^+^ monocytes purified from PB were also treated with rh IL8 and with the conditioned medium of hTERT-MSCs treated with MM1.S exosomes in the presence or not of CXCR1-CXCR2 inhibitor (SB225002). At the end of the culture period, OC differentiation was assessed.

#### OB differentiation

Lastly, in other experimental setting, hTERT-MSCs were used to evaluate the role of MM exosomes on OB differentiation. hTERT-MSCs were treated for 10 and 14 days with exosomes from MM1.S or from MM plasma patients in undifferentiating or osteogenic differentiation medium; the media were changed every 3 days. At the end of the culture period, osteogenic differentiation, exosome uptake, and EGFR activation were assessed.

#### OC differentiation

OC differentiation of human PB CD14^+^ were evaluated after 10 days of culture conditions by the detection of tartrate-resistant acid phosphatase (TRAP) activity, according to the manufacturer’s protocol (Acid Phosphatase, Leukocyte (TRAP) Kit; Sigma–Aldrich, USA) and evaluated by light microscopy. Three independent experiments were performed in triplicate; cells from five different fields were counted for each condition.

### Atomic force microscopy

Fresh cleaved mica was incubated with a vesicle solution diluted in PBS to a final concentration of 30 ng/μl for 15 min at room temperature. Sample was gently rinsed by PBS, and tapping mode atomic force microscopy (AFM) measurements were carried out in liquid by using a Nanowizard III scanning probe microscope (JPK Instruments AG, Germany) equipped with a 15-μm scanner, and AC40 (Bruker) silicon cantilevers (nominal spring constant 0.1 N/m, typical tip radius 10 nm, resonance frequency 55 kHz, scan rate 1.5 Hz, free oscillation amplitude 7 nm).

### Dynamic light scatter

Exosome size distribution was determined by dynamic light scattering (DLS) experiments. Collected MM-exosome patient samples were diluted to avoid inter-particle interaction and placed at 20 °C in a thermostatic cell compartment of a Brookhaven Instruments BI200-SM goniometer, equipped with a Brookhaven BI-9000 correlator and a solid-state laser tuned at 532 nm. Scattered intensity autocorrelation functions were analyzed by using a constrained regularization method or alternatively a gamma distribution [16, 26] in order to determine the size distribution (namely the z-averaged hydrodynamic diameter distribution).

### Uptake of MM-derived exosomes by hTERT-MSCs

MM1.S exosomes were labeled with PKH26 Red Fluorescent Cell Linker Kits (Sigma–Aldrich, USA) according to the supplier’s information. Specifically, exosomes collected after the 100,000×*g* ultracentrifugation were incubated with PKH26 dye, previously diluted in the diluent C solution, for 10 min at room temperature. Labeled exosomes were washed in PBS by ultracentrifugation; the pellets were suspended in low serum medium and incubated with hTERT-MSCs for 3 h. hTERT-MSCs were grown on coverslips coated with COL1A1 (Calbiochem, Darmstadt, Germany) and were treated with 50 μg/ml of exosomes pre-treated or not with anti AREG mAb. hTERT-MSCs were stained with Actin Green (Molecular Probes, Life Technologies, Carlsbad, CA, USA) that binds actin with high affinity. Nuclei were stained with Hoechst (Molecular Probes, Life Technologies, Carlsbad, CA, USA) and analyzed by confocal microscopy. Fluorescence intensity was measured using IMAGE J software (https://imagej.nih.gov/ij//).

### Adhesion assay

Adhesion assay was performed as previously described by our group [27]. Briefly, hTERT-MSCs monolayer was incubated for 48 h with 50 μg/ml of MM1.S exosomes pre-treated or not with anti AREG mAb. After treatments, cells were washed with PBS and MM1.S cells were added for 3.5 h at 37 °C. Adherent cells were stained with hematoxylin/eosin, and each test group was assayed in triplicate; five high-power (400×) fields were counted for each condition.

### Osteolmage bone mineralization assay

The amount of in vitro mineralization of hTERT-MSCs, seeded in 96-well tissue culture plates and treated for 10 and 14 days with exosomes from MM1.S cells in undifferentiating medium or in osteogenic differentiation medium, was evaluated using the Osteolmage Mineralization Assay Kit (Lonza, Walkersville, MD, USA), according to the supplier’s information. Briefly, after each culture time point, media was removed; cells were washed in PBS and fixed. After fixation, cells were washed in the appropriate buffer and the staining reagent added. Mineralization was quantitated on a fluorescent plate reader at a 492/520 nm ratio.

### Flow cytometry

Phosphorylation levels of EGFR in hTERT-MSCs incubated for 48 h with 50 μg/ml of MM1.S exosomes pre-treated or not with anti AREG mAb were determined by flow cytometry. Cells were fixed and permeabilized with Leucoperm kit (AbDSerotec). EGFR- or phospho-EGFR unconjugated primary antibody (Cell Signalling Technology, Lane Danvers, MA, USA) was added; cells were washed, and a FITC secondary antibody was added. Stained cells were analyzed on a FACS Calibur (Becton Dickinson) using Cellquest software.

### Western blot assay

Total proteins from MM1.S cells lysates, MM1.S exosome, conditioned medium of cells deprived of exosomes, patient’s exosomes, and RAW 264.7 lysates were extracted and analyzed by SDS-PAGE followed by western blotting. Antibodies used in the experiments were as follows: anti-EGFR, pEGFR (Cell Signalling Technology, Lane Danvers, MA, USA), anti-AREG (Novus Biologicals), and anti-GAPDH (Santa Cruz Biotechnology, CA, USA).

### RNA extraction and real-time PCR

RNA was extracted using the commercially available IllustraRNAspin Mini Isolation Kit (GE Healthcare, Little Chalfont, Buckinghamshire, UK), according to the manufacturer’s instructions. Total RNA was reverse transcribed to cDNA using the High Capacity cDNA Reverse Transcription Kit (Applied Biosystems, Foster City, CA, USA). RT-QPCR was performed in 48-well plates using the Step-One Real-Time PCR system (Applied Biosystems). For quantitative Sybergreen real-time PCR, reaction was carried out in a total volume of 20 μl containing 2× SYBR Green I Master Mix (Applied Biosystems), 2 μl cDNA, and 300 nM forward and reverse primers. Primer sequences, obtained from Invitrogen (Foster City, CA, USA), were as follows:

- Human GAPDH (5′-ATGGGGAAGGTGAAGGTCG-3′, 5′-GGGTCATTGATGGCAACAATAT-3′)
- Human SNAIL (5′-GCGAGCTGCAGGACTCTAAT-3′, 5′-CCCGCAATGGTCCACAAAAC-3′)
- Human MMP9 (5′-CGCTACCACCTCGAACTTTG-3′, 5′-GCCATTCACGTCGTCCTTAT-3′)
- Human TRAP (5′-GATCCTGGGTGCAGACTTCA-3′, 5′-GCGCTTGGAGATCTTAGAGT-3′)
- Human CTSK (5′-ACCGGGGTATTGACTCTGAA-3′, 5′-GAGGTCAGGCTTGCATCAAT-3′)
- Human IL8 (5′-GAATGGGTTTGCTAGAATGTGATA-3′, 5′-CAGACTAGGGTTGCCAGATTTAAC-3′)
- Human ALP (5′-ACCGGGGTATTGACTCTGAA-3′, 5′-GAGGTCAGGCTTGCATCAAT-3′)
- Human OCN (5′-GAGGGCAATAAGGTAGTGAA-3′, 5′-CATAGATGCGTTTGTAGGC-3′)
- Human COL1A1 (5′-AAGGTGTTGTGCGATGACGTG-3′, 5′-CACGTCATCGCACAACACCTT-3′)
- Human OPG (5′-GGCAACACAGCTCACAAGAA-3′, 5′-CTGGGTTTGCATGCCTTTAT-3′)
- Mouse Gapdh (5′-CCCAGAAGACTGTGGATGG-3′, 5′-CAGATTGGGGGTAGGAACAC-3′)
- Mouse Trap (5′-GCGACCATTGTTAGCCACATACG-3′, 5′-CGTTGATGTCGCACAGAGGGAT-3′)
- Mouse Ctsk (5′-GCGTTGTTCTTATTCCGAGC-3′, 5′-CAGCAGAGGTGTGTACTATG-3′)
- Mouse Mmp9 (5′-GCTGACTACGATAAGGACGGCA-3′, 5′-GCGGCCCTCAAAGATGAACGG-3′)
- Mouse Snail (5′-GCGAGCTGCAGGACTCTAAT-3′, 5′-CCCGCAATGGTCCACAAAAC-3′).

Human RANKL gene expression was assessed using the TaqMan Gene Expression Assay (Life Technologies, Milan, Italy).

Real-time PCR was performed in triplicates for each data point. Relative changes in gene expression between control and treated samples were determined using the ΔΔCt method. Levels of the target transcript were normalized to a GAPDH endogenous control, constantly expressed in all samples (ΔCt). For ΔΔCt values, additional subtractions were performed between treated samples and control ΔCt values. Final values were expressed as fold of induction.

### ELISA assay

MMP-9 levels were quantified by Human MMP-9 ELISA assays (Invitrogen) for CD14^+^ monocytes and with mouse total MMP9–enzyme-linked immunosorbent assays (R&D Systems, Minneapolis, MN, USA) for RAW 264.7 cells according to the manufacturer’s protocol. Furthermore, the levels of ALP and IL8 secreted by hTERT-MSCs were quantified respectively by ALP ELISA assay (Cloud-Clone Corp ELISA KIT assay, Cloud-Clone Corp. Houston, TX, USA) and Human IL8 ELISA assay (R&D Systems, Minneapolis, MN) according to the manufacturer’s protocol.

### Statistical analysis

Data are expressed as means ± SD of three independent experiments. Statistical analysis was done with two-tailed unpaired *t* test using Graphpad Prism. Differences were considered significant when *p* ≤ 0.05.

## Results

### The EGFR ligand AREG is expressed by MM cells and enriched in exosomes

**Fig. 1:**
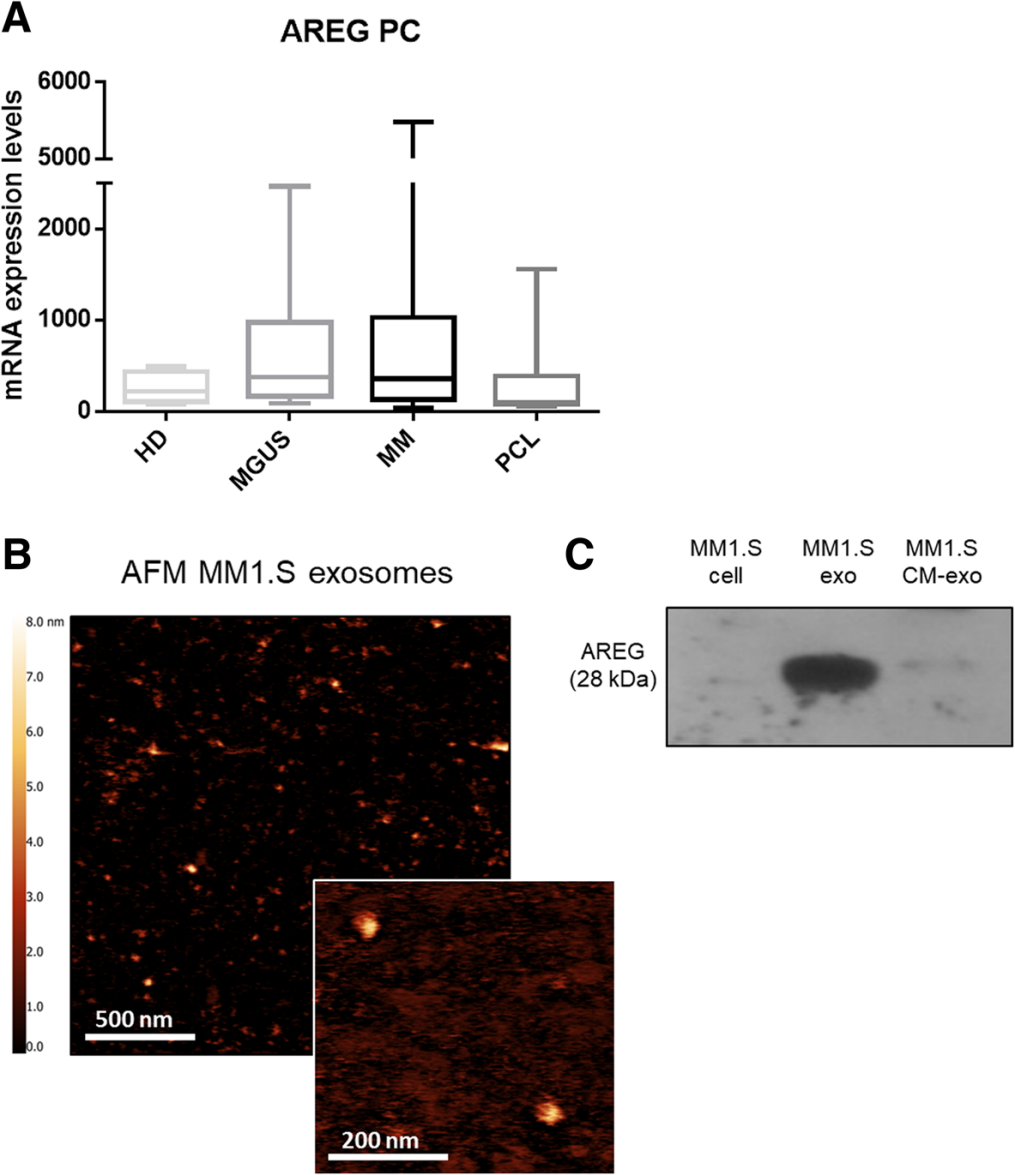
**a** Box plot represents the median level of AREG expression of 11 monoclonal gammopathy of undetermined significance (MGUS), 133 MM patients at diagnosis, and 9 plasma cell leukemia (PCL) (GSE16122). **b** Representative AFM image of MM1.S exosomes. **c** Total proteins were extracted from MM1.S cells, MM1.S exosomes (exo), and exosome-deprived conditioned medium (cm-exo) and were subjected to western blot analysis with an antibody against AREG

By analyzing the mRNA expression level of EGFR ligands in a published dataset (accession number GSE16122), we found that CD138^+^ cells expressed AREG mRNA at variable levels among the different type of monoclonal gammopathies (Fig. 1a). Consistently, we found that HMCLs, as well as MM1.S-derived exosomes, expressed AREG mRNA (Additional file 1: Figure S1).

Exosomes were then isolated from one HMCL MM1.S and characterized by the atomic force microscope (AFM) in order to confirm that we are working with vesicles of about 80 nm (Fig. 1b). We found that AREG was specifically enriched in exosomes as confirmed by its low abundance in MM1.S cells and in the exosomes-deprived conditioned medium (Fig. 1c).

### MM1.S-exosomes induce the activation of EGRF pathway in OC progenitor cells

**Fig. 2:**
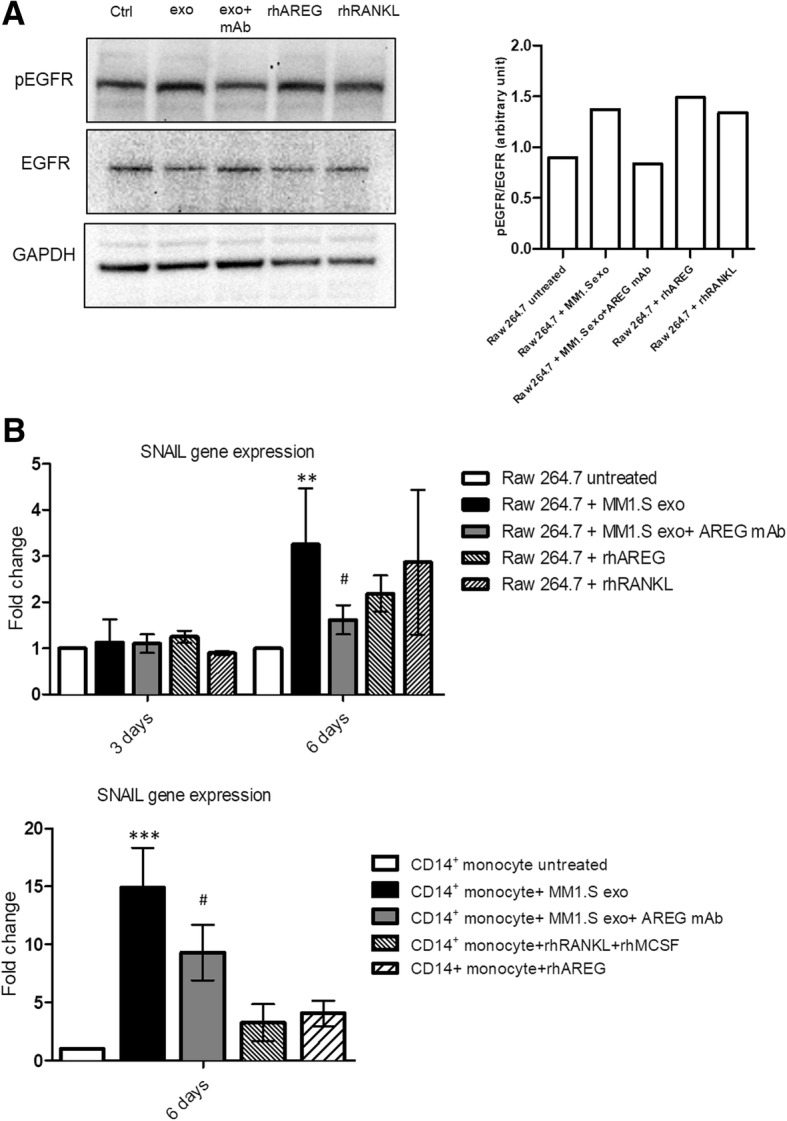
**a** Western blotting analysis of pEGFR and EGFR in whole lysates of RAW 264.7 cells incubated, for 6 days, with MM1.S derived exosomes (50 μg/ml) treated or not with anti-AREG mAb (50 μg/ml), with rhAREG (50 μg/ml) and rhRANKL (25 μg/ml). The histogram on the right represents the ratio pEGFR/EGFR, based on densitometric analysis normalized versus GAPDH, used as loading control. **b** Evaluation by quantitative real-time PCR of mRNA expression of SNAIL in RAW 264.7 incubated, for 3 and 6 days, with MM1.S-derived exosomes (50 μg/ml) treated or not with anti-AREG mAb (50 μg/ml), rhRANKL (25 μg/ml), and rhAREG (50 μg/ml). Human PB CD14^+^ cells incubated for 6 days in osteoclastogenic medium (rhRANKL 25 ng/ml and MCSF 25 ng/ml), with MM1.S derived exosomes (50 μg/ml) treated or not with anti-AREG mAb (50 μg/ml). *Exo vs untreated (**p ≤ 0.01; ***p ≤ 0.001); ^**#**^Exo+AREG mAb vs Exo (^#^*p* ≤ 0.05)

We next investigated whether MM1.S-exosomes treatment induced the activation of EGFR pathway in OC progenitors. RAW 264.7 cells treated with MM1.S cell line exosomes under osteoclastogenic conditions showed an increase in the phosphorylation of EGFR (Fig. 2a). The treatment with exosomes derived from MM1.S cells pretreated with anti-AREG mAb reduces the phosphorylation of EGFR. Subsequently, we found that the 6day treatment with MM1.S-derived exosomes induced a significant increase in the mRNA expression level of SNAIL, a downstream target of EGFR, both in RAW 264.7 (Fig. 2b, upper panel) and in pre-osteoclast human CD14^+^ (Fig. 2b, lower panel). The presence of anti-AREG mAb reverted this effect (Fig. 2b).

### AREG-enriched MM cell-derived exosomes induced OC differentiation

**Fig. 3:**
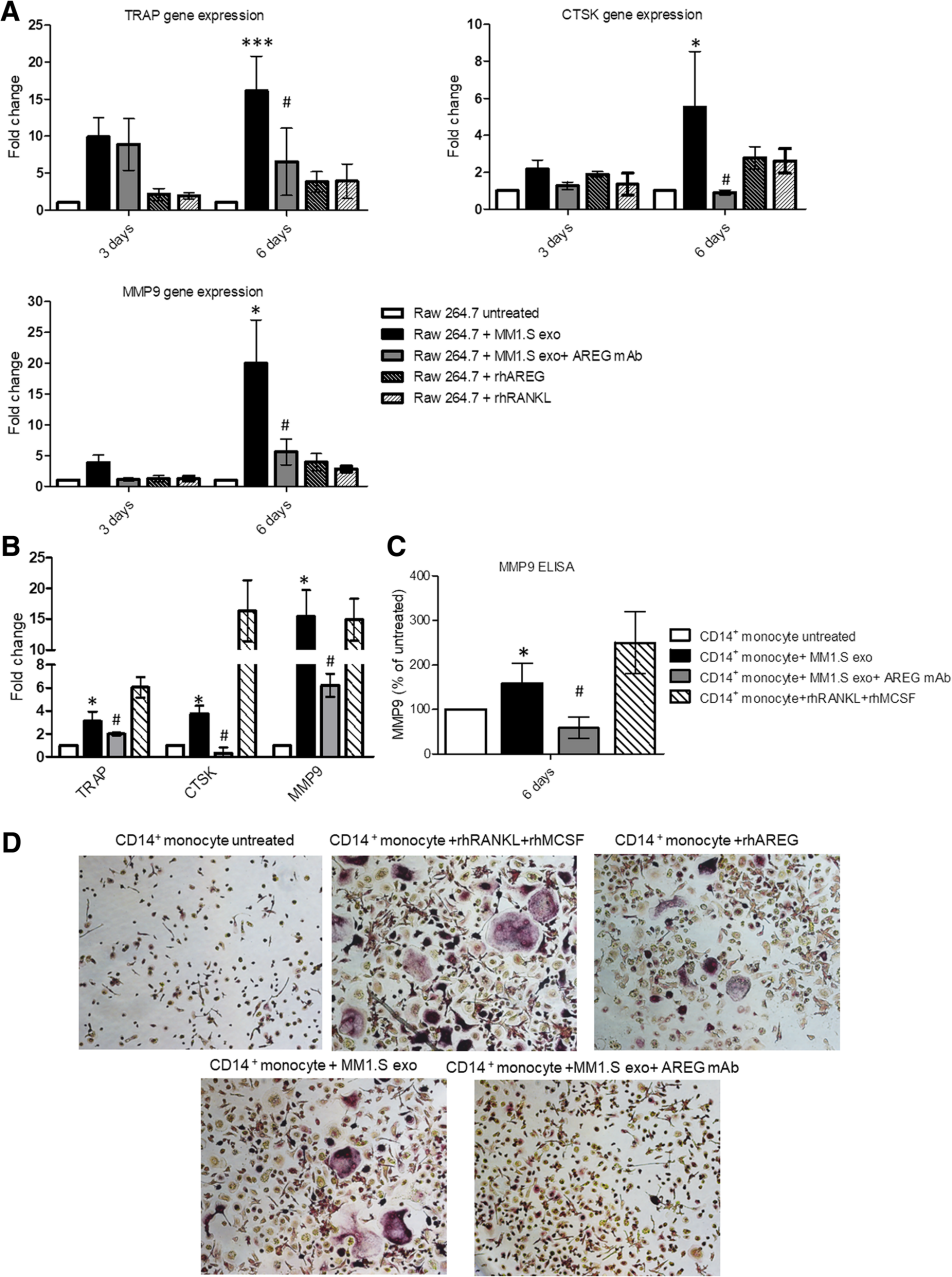
Evaluation by quantitative real-time PCR of mRNA expression of TRAP, CTSK, and MMP9 in **a** RAW 264.7 incubated, for 3 and 6 days, with MM1.S-derived exosomes (50 μg/ml) treated or not with anti-AREG mAb (50 μg/ml), rhRANKL (25 μg/ml), and rhAREG (50 μg/ml). **b** Human PB CD14^+^ cells incubated, for 6 days in osteoclastogenic medium (rhRANKL 25 ng/ml and MCSF 25 ng/m), with MM1.S derived exosomes (50 μg/ml) treated or not with anti-AREG mAb (50 μg/ml). *Exo vs untreated (*p ≤ 0.05; ***p ≤ 0.001); ^**#**^Exo+AREG mAb vs Exo (^#^*p* ≤ 0.05). **c** MMP9 protein level was measured by ELISA in the conditioned medium of Human PB CD14^+^ cells incubated, for 6 days in osteoclastogenic medium (rhRANKL 25 ng/ml and MCSF 25 ng/m), with MM1.S-derived exosomes (50 μg/ml) treated or not with anti-AREG mAb (50 μg/ml). *Exo vs untreated (*p ≤ 0.05); ^**#**^Exo+AREG mAb vs Exo (^#^*p* ≤ 0.05). **d** Trap staining of Human PB CD14^+^ seeded in 96 well plates in presence or absence of MM1.S-derived exosomes (50 μg/ml) treated or not with mAb AREG and rhAREG anti-AREG mAb (50 μg/ml) for 6 days

Raimondi et al. demonstrated that MM-derived exosomes directly induce the expression of OC-specific markers [16]. We confirmed that the treatment with MM-derived exosomes from MM1.S increases OC-specific markers, such as TRAP, CTSK, and MMP9 at mRNA level both in RAW 264.7 (Fig. 3a) and PB human CD14^+^ cells (Fig. 3b). This effect was confirmed at protein level for MMP-9 (Fig. 3c) and by TRAP staining (Fig. 3d). The pro-osteoclastogenic effect of MM-derived exosomes was significantly abrogated by the pre-treatment with anti-AREG mAb (Fig. 3a–d) suggesting a direct effect of MM exosomes-derived AREG on OC differentiation.

**Fig. 4:**
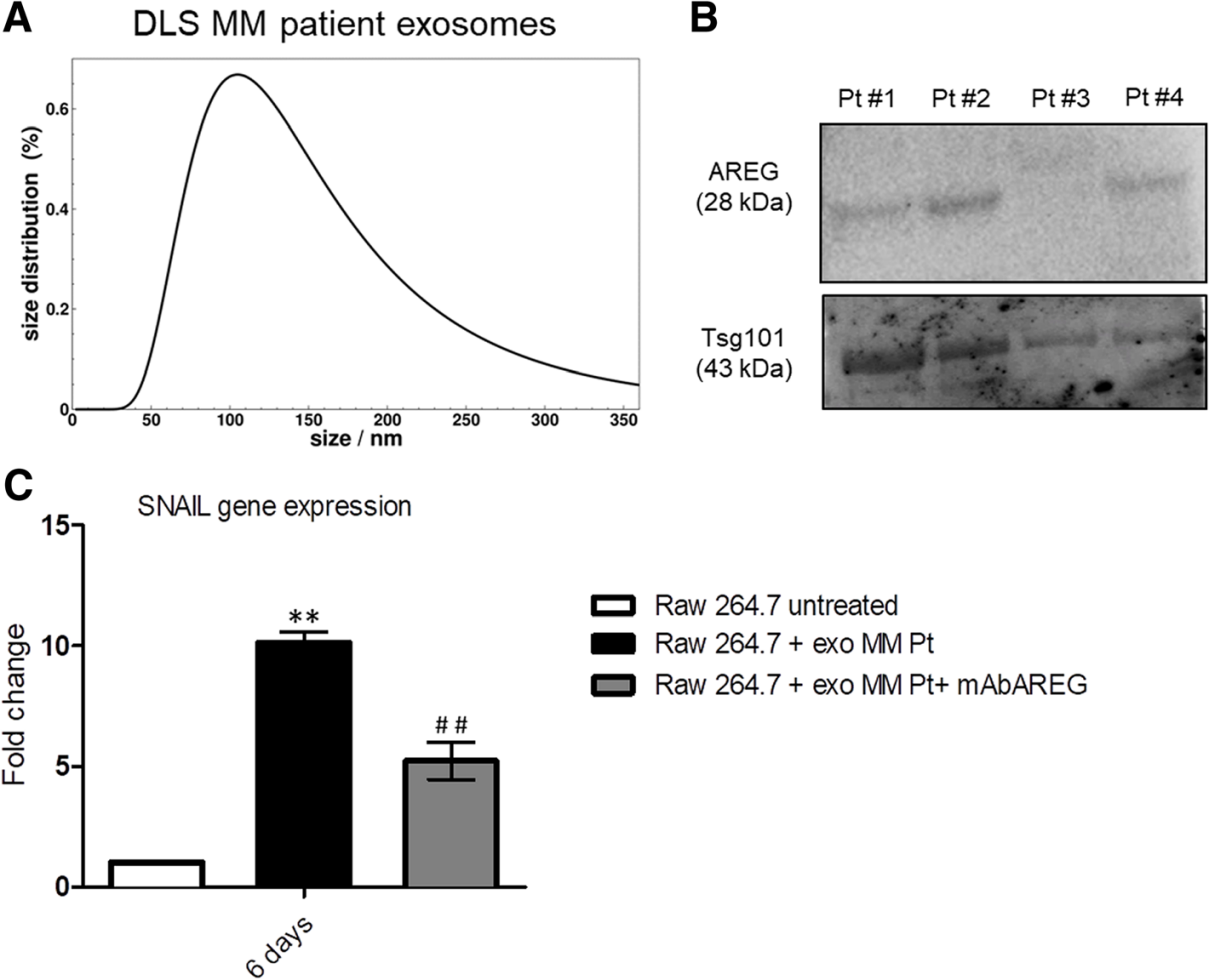
**a** MM patient exosomes size distribution was determined by DLS analysis. **b** Total proteins were extracted from exosomes isolated from the BM plasma of MM patients and were subjected to western blot analysis with antibody against AREG and Tsg101. The table below indicates the clinical information of the four MM patients analyzed. **c** Evaluation by quantitative real-time PCR of mRNA expression of SNAIL in Raw 264.7 cells incubated with exosomes from MM patients (50 μg/ml) treated or not with anti-AREG mAb (50 μg/ml). *Exo vs untreated (**p ≤ 0.01); ^**#**^Exo+AREG mAb vs Exo (^##^*p* ≤ 0.01)

**Fig. 5:**
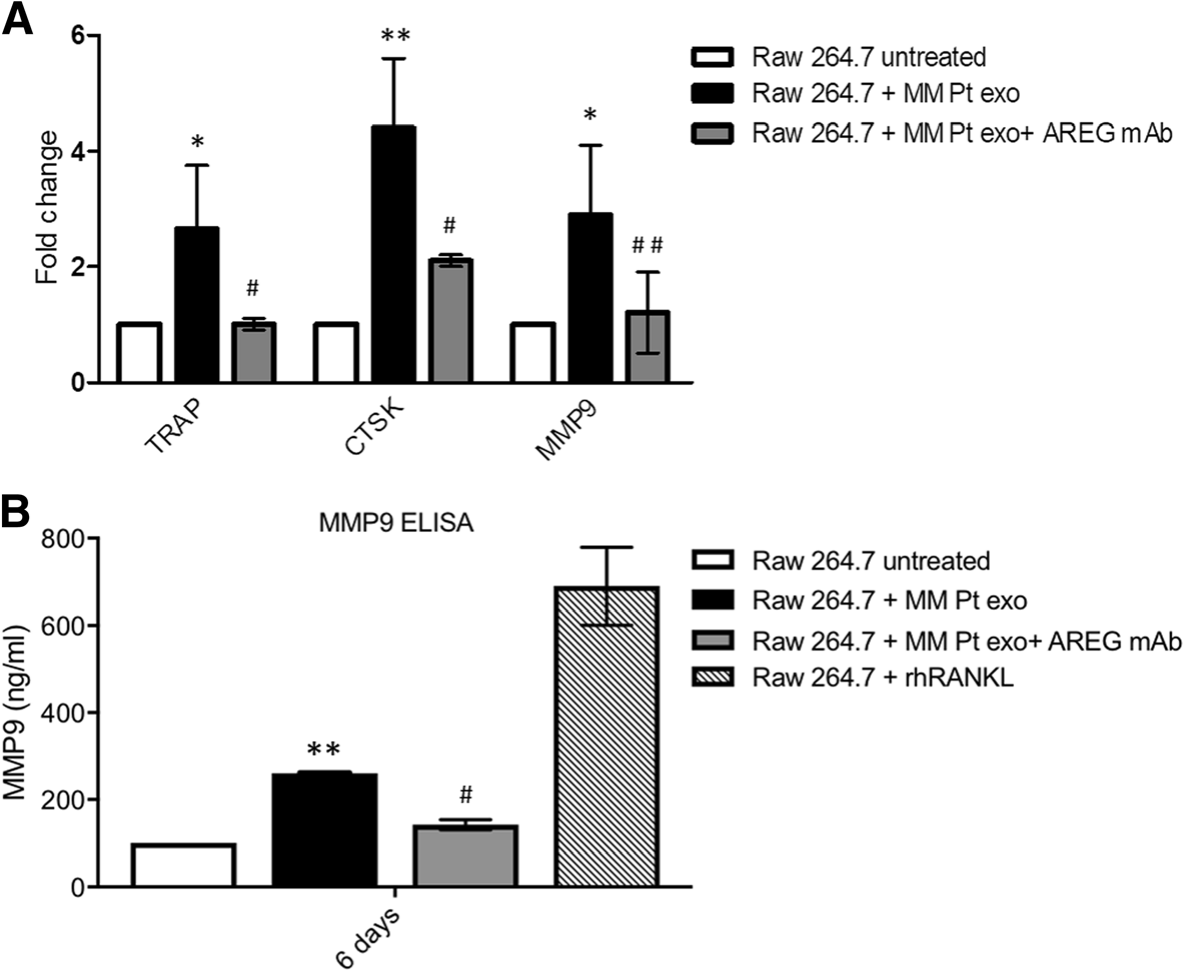
**a** Evaluation by quantitative real-time PCR of mRNA expression of TRAP, CTSK and MMP9 in Raw 264.7 cells incubated with exosomes from MM patients (50 μg/ml) treated or not with anti-AREG mAb (50 μg/ml). **b** MMP9 protein level was measured in the conditioned medium of Raw 264.7 cells incubated, for 6 days, with exosomes from MM patients (50 μg/ml) treated or not with anti-AREG mAb (50 μg/ml), rhRANKL (25 μg/ml), and rhAREG (50 μg/ml). *Exo vs untreated (*p ≤ 0.05; **p ≤ 0.01); ^**#**^Exo+AREG mAb vs Exo (^#^*p* ≤ 0.05; ^##^*p* ≤ 0.01)

On the basis of these data, we investigated whether ex vivo exosomes from MM patients deliver the EGFR ligand. Exosomes were isolated from BM aspirates of MM patients. The vesicles were analyzed with DLS, showing a clear distribution with peak at about 100 nm (Fig. 4a) and with western blot for TSG101 (Fig. 4b). AREG was enriched in the exosomes obtained from three out of four patients, thus confirming the exosomal packaging of the ligand. As observed with exosomes from MM1.S cell line, we found that also patient-derived exosomes increase the expression of SNAIL in pre-OCs, while the presence of anti-AREG mAb abolished this effect (Fig. 4c). Similarly, the pro-osteoclastogenic effects of exosomes obtained from MM patients were abrogated by the pre-treatment with the anti-AREG mAb at mRNA (Fig. 5a) and protein level as shown for MMP9 (Fig. 5b). Overall, these data indicate that the EGFR ligand AREG is packed into MM-derived exosomes and directly involved in OC differentiation.

### MM-derived exosomes are internalized into human BM mesenchymal cells blocking osteogenic differentiation and increasing the release of the pro-osteoclastogenic cytokines through the activation of EGFR pathway

**Fig. 6:**
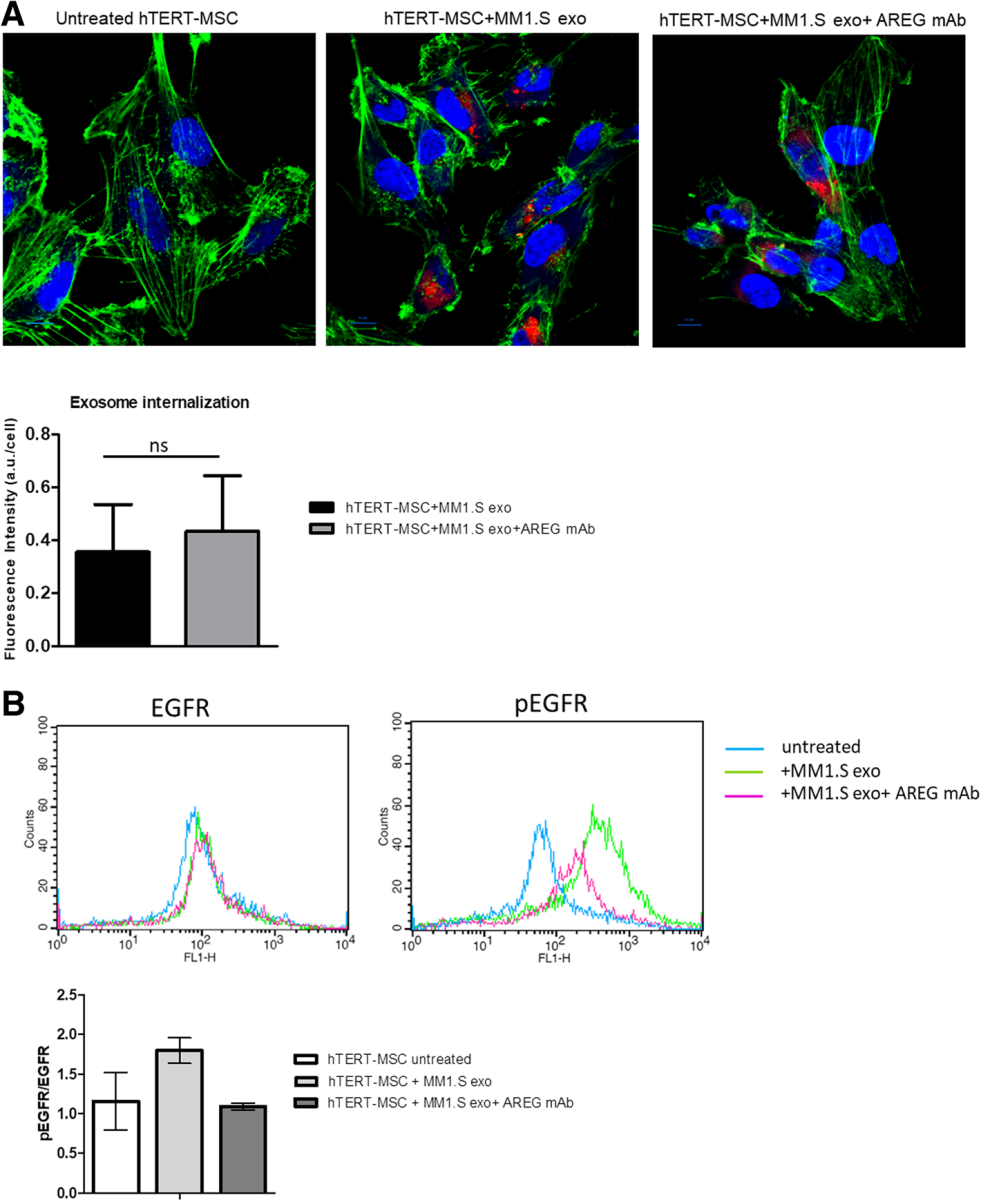
**a** Analysis at confocal microscopy of hTERT-MSCs treated for 4 h with MM1.S exosomes pretreated or not with mAb AREG, compared with untreated hTERT-MSCs (Ctrl). hTERT-MSCs were stained with phalloidin Alexa Fluor (green), nuclear counterstaining was performed using Hoescht (blue), and exosomes were labeled with PKH26 (red); histogram shows fluorescence intensity expressed as ratio between a.u. and number of hTERT-MSCs treated with MM1.S exosomes and MM1.S exosomes pretreated with mAb AREG. **b** Levels of EGFR and phospho-EGFR were determined by FACS analysis in hTERT-MSCs after 48 h treatment with MM1.S exosomes pretreated or not with mAb AREG

To further investigate the mechanism by which AREG-enriched exosomes from MM cells are involved in MM-induced alteration of bone remodeling, we evaluated the effect of exosomes on hTERT-MSCs. We demonstrated that MM1.S-derived exosomes are internalized into hTERT-MSCs independently by the neutralization of AREG (Fig. 6a). Exosomes internalization by hTERT-MSCs induced the activation of EGFR pathway as demonstrated by the increase of the tyrosine kinase receptor phosphorylation. EGFR activation was blocked by the treatment of anti-AREG mAb (Fig. 6b).

**Fig. 7:**
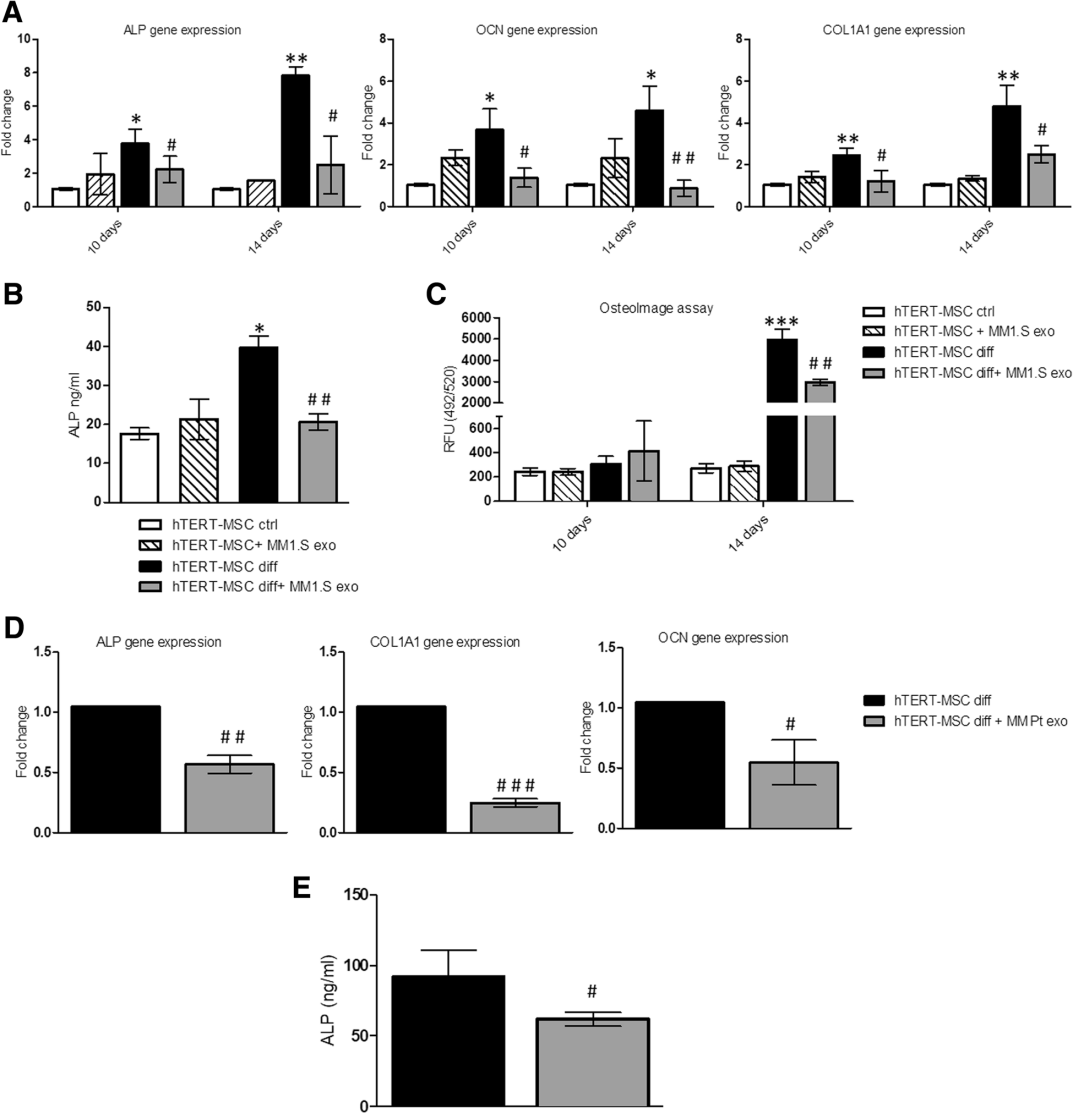
**a** Evaluation by quantitative real-time PCR of mRNA expression of ALP, OCN, and COL1A1 in hTERT-MSC treated for 10 and 14 days with MM1.S exosomes under undifferentiating medium (ctrl) or in osteogenic differentiation medium (diff). **b** ALP protein release was evaluated by ELISA assay in the conditioned medium of hTERT-MSC treated for 14 days with MM1.S exosomes under undifferentiating medium (ctrl) or in osteogenic differentiation medium (diff). **c** Quantification of in vitro osteoblast mineralization in the hTERT-MSC treated for 10 and 14 days with MM1.S exosomes under undifferentiating medium (ctrl) or in osteogenic differentiation medium (diff) was evaluated using OsteoImage Mineralization Assay Kit. Values are expressed as fluorescence units (RFU; 492 nm excitation/520 nm emission wavelengths). *Diff vs ctrl (*p ≤ 0.05; **p ≤ 0.01; ***p ≤ 0.001); ^**#**^Diff+ Exo vs diff (^#^*p* ≤ 0.05; ^##^*p* ≤ 0.01). **d** Evaluation by quantitative real-time PCR of mRNA expression of ALP, OCN, and COL1A1 in hTERT-MSC treated for 14 days with exosomes isolated from BM plasma of three MM patients in osteogenic differentiation medium (diff). **e** ALP protein release was evaluated by ELISA assay in the conditioned medium of hTERT-MSC treated for 14 days with exosomes isolated from BM plasma of three MM patients in osteogenic differentiation medium (diff). ^**#**^Diff+ Exo vs diff (^#^*p* ≤ 0.05; ^##^*p* ≤ 0.01; ^###^*p* ≤ 0.001)

Interestingly, under osteogenic conditions, the treatment of hTERT-MSCs with MM1.S-derived exosomes for 14 days reduces the mRNA levels of OB differentiation markers (Fig. 7a). In addition to gene expression, the ability of MM exosomes to inhibit ALP release was evaluated at the protein level (Fig. 7b). OB differentiation is characterized by the formation of mineralized nodules. Therefore, we performed an in vitro mineralization assay in order to functionally evaluate the effect of MM exosomes on bone-like nodules deposited by cells. As shown in Fig. 7c, 14 days of treatment of hTERT-MSCs with MM1.S exosomes under osteogenic conditions reduces the formation of mineralized nodules. No differences were observed in exosomes-treated MSCs maintained into an undifferentiated state. To further confirm the observed exosomes-mediated inhibition of OB differentiation, we performed a qRT-PCR of cells treated with exosomes from the BM aspirates of MM patients. Accordingly, 14day treatment of hTERT-MSCs under osteogenic conditions decreases OB differentiation marker at the mRNA (Fig. 7d) and protein level (Fig. 7e).

**Fig. 8:**
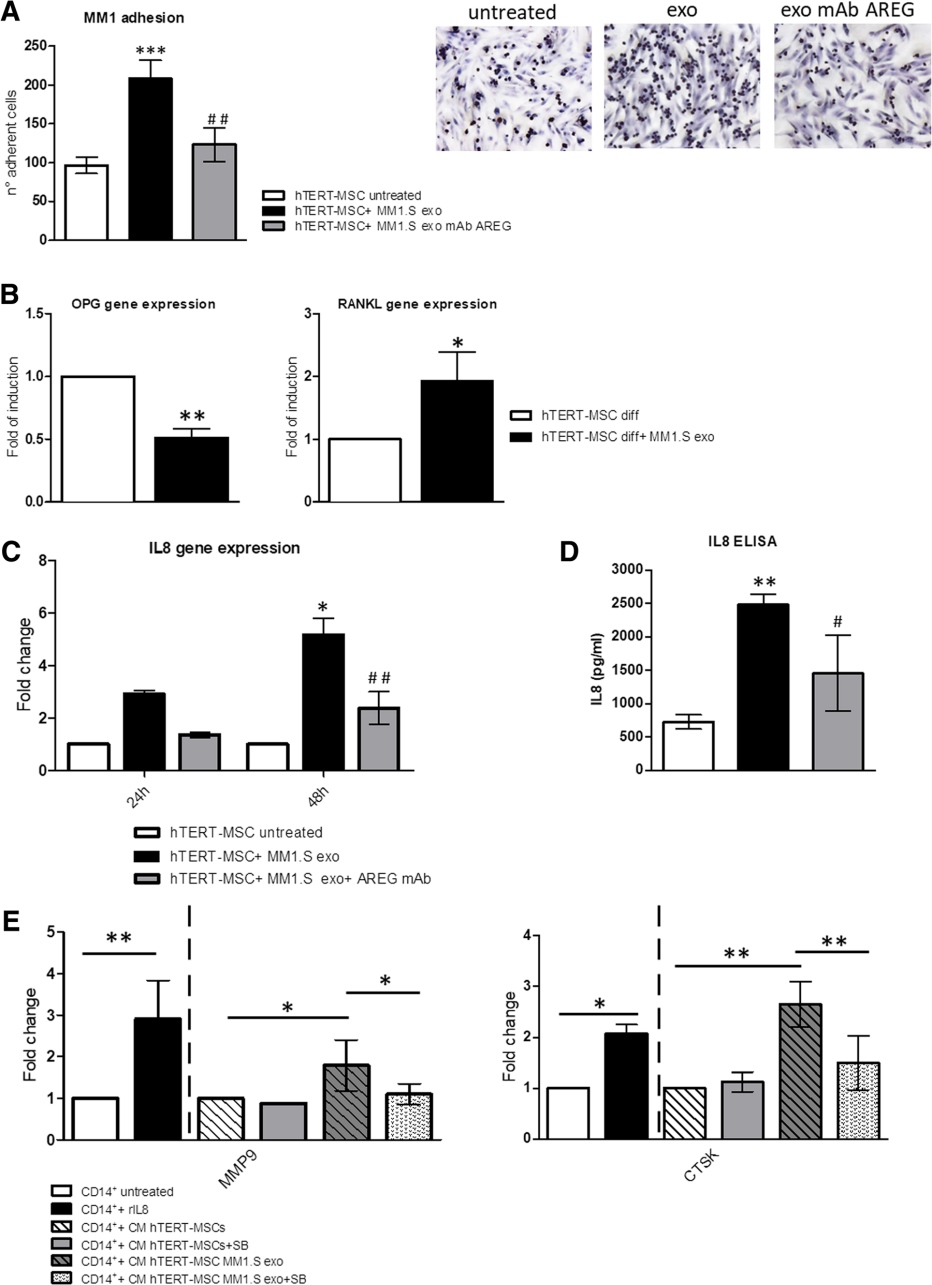
**a** Adhesion assay of MM1.S exo on hTERT-MSCs: pre-treatment of hTERT-MSCs cells with MM1.S exo for 48 h increases MM1.S cell adhesion to mesenchymal cells. Treatment with exosomes pretreated with anti-AREG mAb reduces this effect. Right panel: a representative phase contrast micrograph showing the adhesion of MM1.S cells to exosome-treated hTERT-MSCs monolayer. **b** Evaluation by quantitative Real Time PCR of mRNA expression of OPG and RANKL in hTERT-MSC treated for 14 days with MM1.S exosomes in osteogenic differentiation medium (diff) (*p ≤ 0.05; **p ≤ 0.01). **c** IL8 mRNA expression was evaluated by real-time PCR in hTERT-MSCs treated for 24 or 48 h with MM1.S exosomes pretreated or not with anti-AREG mAb for 24 and 48 h. **d** IL8 protein release was evaluated by ELISA assay in the conditioned medium of hTERT-MSCs monolayer after 48 h treatment with MM1.S exosomes pretreated or not with anti-AREG mAb. *Exo vs untreated (*p ≤ 0.05; **p ≤ 0.01; ***p ≤ 0.001); ^**#**^Exo+AREG mAb vs Exo (^#^*p* ≤ 0.05; ^##^*p* ≤ 0.01). **e** Evaluation by quantitative real-time PCR of mRNA expression of CTSK and MMP9 in CD14^+^ monocytes untreated or treated for 6 days with rIL8, with the conditioned medium of BMMSC cells treated with MM1.S exosomes with or without SB225002 (*p ≤ 0.05; **p ≤ 0.01)

Finally, we found that the treatment of hTERT-MSCs with exosomes increases the adhesion of MM1.S cells to the mesenchymal monolayer and that this effect is abrogated by the presence of the anti-AREG mAb (Fig. 8a). Moreover, we found that exosomes are able to reduce OPG mRNA and to increase RANKL mRNA levels by hTERT-MSCs (Fig. 8b). This effect was not inhibited by the pre-treatment with blocking anti-AREG Ab (data not shown) suggesting the lack of a direct effect of AREG on RANKL/OPG expression.

Interestingly, a significant increase in the production of the pro-osteoclastogenic cytokine IL8 by hTERT-MSCs was observed at both mRNA (Fig. 8c) and protein level at 24 and 48 h (Fig. 8d). These effects were abrogated by the pre-treatment with anti-AREG mAb (Fig. 8c, d).

To correlate the increased expression and secretion of IL8 from exosomes-treated MSCs with the exosomes-dependent increase of OC function, we co-treated human CD14^+^ with (i) recombinant IL8 (rIL8) and (ii) with the conditioned medium of hTERT-MSCs treated with MM1.S exosomes, in the presence or absence of the IL8 receptor (CXCR1–2) inhibitor, SB225002 (SB). The treatment with rIL8 induced the expression of MMP9 and CTSK mRNA (Fig. 8e). Consistently, the treatment with conditioned medium of hTERT-MSCs pre-treated with MM exosomes increased the expression of the osteoclastogenic markers. This effect was abrogated by the use of SB (Fig. 8e) thus confirming the role of IL8 released by MSCs after exosomes stimulation, in the activation of OC differentiation.

## Discussion

In this study, we focus on unveiling the molecular mechanism by which MM exosomes are able to affect OC differentiation. We demonstrate that the EGFR ligand AREG is packed into exosomes from MM cell line as well as from the BM aspirates of patients and that its presence is responsible for the exosome-induced osteoclastogenesis. The activation of the EGFR pathway has been correlated to metastatic bone diseases and in particular to the increased bone resorption observed in these tumors [28, 29]. In particular, in breast cancer, Mercatali et al. showed that the crosstalk between MSCs and cancer cells promoted osteoclastogenesis by stimulating RANK and EGFR signaling pathways [30]. Furthermore, EGFR deficiency impaired OC recruitment in EGFR-deficient mice [31]. In MM, Mahtouk and colleagues showed that, among the EGFR ligands, AREG is significantly over-expressed by MM cells as compared to normal PCs and that it is able to stimulate cell growth [32]. We have previously shown, consistent with others, that cancer exosomes contain the EGFR ligands, including AREG, suggesting that the EGFR system contributes to the exosome-mediated communication within the tumor microenvironment [33–35]. For example, Taverna et al. demonstrated that non-small cell lung cancer-derived exosomes, containing AREG, induce EGFR pathway activation in pre-OCs leading to the increased expression of RANKL [34]. Here, we found that AREG is abundantly present in MM exosomes partially explaining the previously observed exosome-mediated increase of OC function in MM [16].

Since the presence of AREG is directly responsible for the activation of OC function in exosome-treated pre-OCs, we further assessed whether the presence of the ligand in the exosomes was able to modulate MSC phenotype and to activate OC formation indirectly through MSCs. We found that the treatment of MSCs with MM exosomes increased the release of IL8 in the conditioned medium, while AREG depletion abrogated the effect. Similarly, our recent data show that the ligand–receptor interaction between AREG produced by leukemic cells, and EGFR by BM stromal cells, modulates leukemic and stromal cells bidirectional crosstalk [35]. In addition, in chronic myeloid leukemia model, we have previously shown that AREG is involved in the activation of EGFR downstream signaling in mesenchymal stromal cells leading to the expression and release of IL8 [27].

IL8 is responsible for the increased osteolysis observed in metastatic bone disease [20] and that its release, following the interaction between MM cells and human MSCs, contributed to in vitro OC formation [21]. Here, we found that exosomes, through the activation of the EGFR pathway, may also indirectly contribute to the induction of osteoclastogenesis by promoting the release of IL8 by MSCs; in fact, IL8-enriched conditioned medium induces the expression of OC-specific markers in human pre-OCs.

Based on our data showing that MM-derived exosomes block osteogenic differentiation of MSCs, it is conceivable to hypotheses that MM exosomes contribute to increase the number of undifferentiated MSCs and consequently the production of pro-osteoclastogenic cytokines as IL8 [21] and RANKL [36]. Accordingly, we show that MM-derived exosomes increased the RANKL mRNA expression and decreased that of OPG. Clearly, this effect can be involved in the indirect pro-osteoclastogenic effect of MM-derived exosomes. However, anti-AREG mAb did not significantly affect RANKL/OPG expression suggesting that the effects of exosomes in reducing OPG mRNA and increase RANKL is regulated by the exosome-mediated increase in cell adhesion. Giuliani et al. demonstrated that the cell-to-cell contact between myeloma cells and BM MSCs by the integrin Very Late Antigen**-**4 (VLA-4) affect the RANKL/OPG ratio a favor of RANKL [36]. We hypothesize that the changes in RANKL/OPG production may be induced by the exosomes-induced increased adhesion of MM cells to mesenchymal cells.

Moreover, exosomes may contribute to the block of bone formation process through the activation of EGFR pathway. In a recent study, Kumar and colleagues in vivo demonstrated that exosomes from acute myeloid leukemia modulate the BM niche; in particular, authors showed that exosomes suppress osteogenic differentiation of mesenchymal stromal progenitors [37]. Other authors observed that MM cells-derived exosome contain the lncRNA RUNX2-AS1 which is responsible for the decreased expression of RUNX2 in MSCs, leading to the osteogenesis suppression [38]. Although in this study authors identified one of the molecular interactor of the exosome-mediated osteogenic inhibition, further studies need to be conducted in order to characterize MM exosome content and fully understand how MM exosomes contribute to the uncoupled bone remodeling by the inhibiting bone formation.

The observation that MM cell-derived exosomes induced the activation of EGFR pathway in both OC progenitors and in MSCs suggests the possibility to use EGFR inhibitors such as erlotinib and gefitinib to impair the cross talk between MM cells and the bone microenvironment and potentially the development of bone lesions. Consistently, it was reported that erlotinib inhibits osteolytic bone invasion of non-small lung cancer [39] and that gefitinib inhibits the ability of MSC to induce OC differentiation [40].

## Conclusions

In conclusion, our data indicate that MM-derived exosomes could be responsible for the uncoupled bone remodeling increasing OC differentiation both directly and indirectly through, at least in part, the release of IL8 by MSCs (Additional file 2: Figure S2). Thus, AREG packed into MM-derived exosomes may represent a potential new player in MM-induced osteoclastogenesis.

## Additional files

Additional file 1: **Figure S1.** Evaluation by quantitative Real Time PCR of mRNA expression of AREG in HMCLs (A) and in MM1.S cells and exosomes (B). (TIFF 2501 kb)

Additional file 2: **Figure S2.** Schematic representation of the role of MM-exosomes in bone microenvironment. (TIF 3372 kb)

## Abbreviations

AFM: Atomic force microscopy
AREG: Amphiregulin
BM: Bone marrow
cDNA: Complementary DNA
CTSK: Cathepsin K
DLS: Dynamic light scatter
EGFR: Epidermal growth factor receptor ligands
FBS: Fetal bovine serum
HMCL: Human myeloma cell line
IL8: Interleukin-8
mAb: Monoclonal antibody
MM: Multiple myeloma
MMP9: Matrix metallopeptidase 9
MSCs: Mesenchymal stromal cells
OB: Osteoblast
OC: Osteoclast
OPG: Osteoprotegerin
PBMC: Peripheral mononuclear cell
PC: Plasma cell
RANKL: Nuclear factor kappa-Β ligand
TRAP: Tartrate-resistant acid phosphatase
