# Supplementary figures and images for: Multiple myeloma-derived exosomes are enriched of amphiregulin (AREG) and activate the epidermal growth factor pathway in the bone microenvironment leading to osteoclastogenesis
Source: J Hematol Oncol. 2019 Jan 8;12:2. doi: 10.1186/s13045-018-0689-y (PMC6325886; doi:10.1186/s13045-018-0689-y)

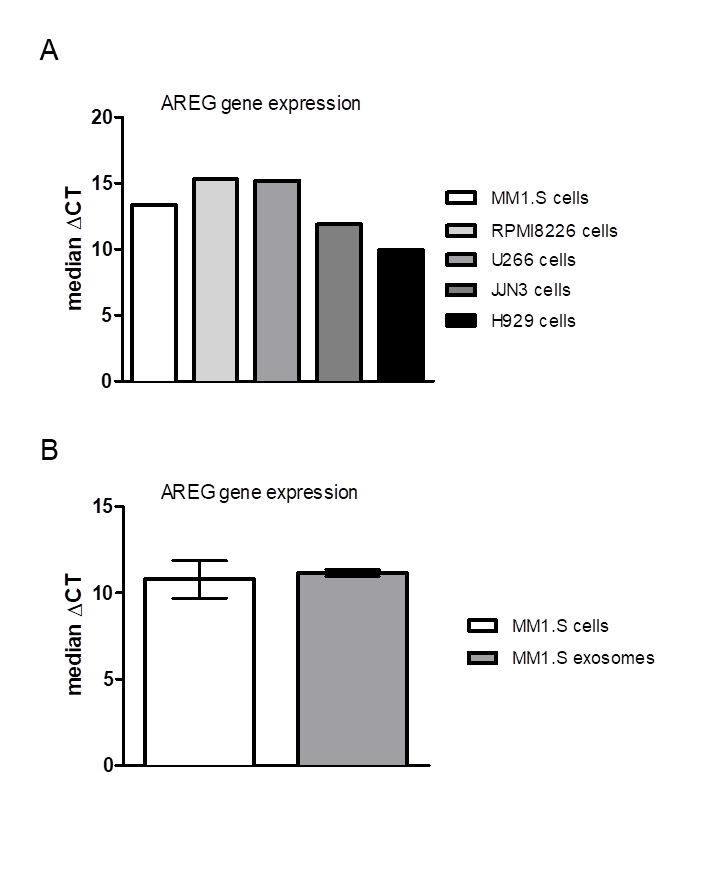

Supplement: Supplementary file 1 — Figure S1. Evaluation by quantitative Real Time PCR of mRNA expression of AREG in HMCLs (A) and in MM1.S cells and exosomes (B). (TIFF 2501 kb) [file 13045_2018_689_MOESM1_ESM.tiff]

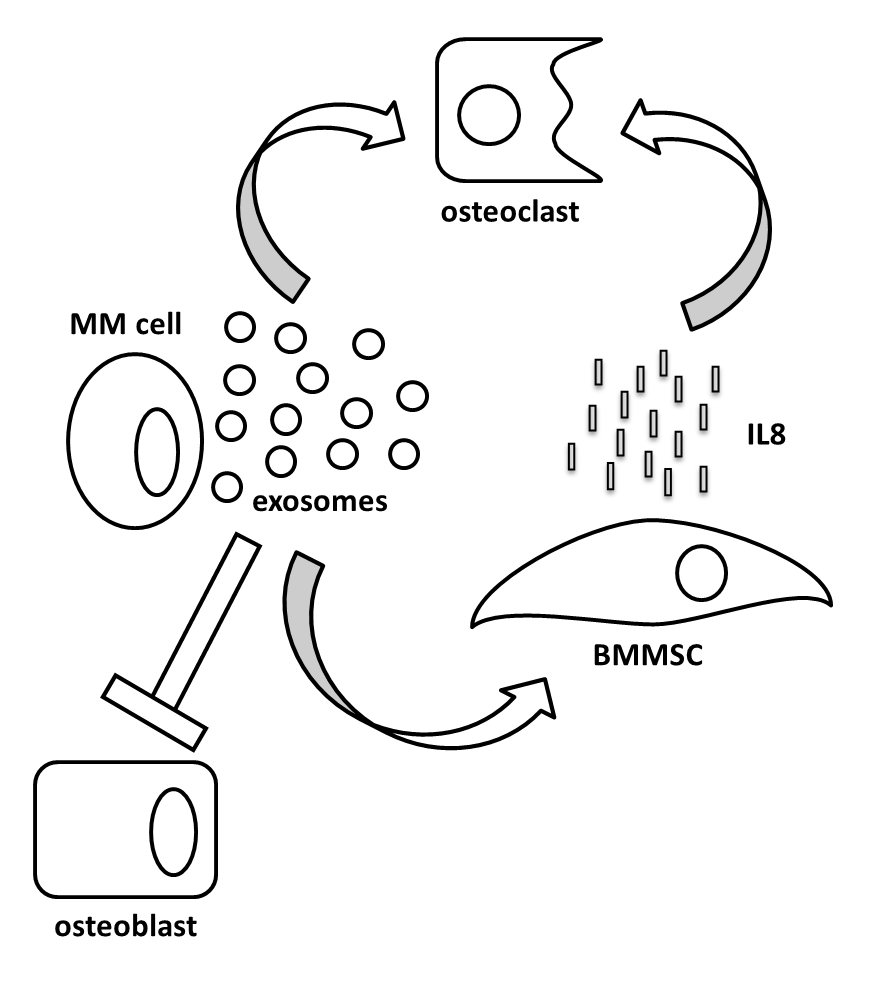

Supplement: Supplementary file 2 — Figure S2. Schematic representation of the role of MM-exosomes in bone microenvironment. (TIF 3372 kb) [file 13045_2018_689_MOESM2_ESM.tif]
